# Medicare Advantage Enrollment Following the 21st Century Cures Act in Adults With End-Stage Renal Disease

**DOI:** 10.1001/jamanetworkopen.2024.32772

**Published:** 2024-09-12

**Authors:** Kevin H. Nguyen, Eunhae G. Oh, David J. Meyers, Maricruz Rivera-Hernandez, Daeho Kim, Rajnish Mehrotra, Amal N. Trivedi

**Affiliations:** 1 Department of Health Law, Policy, and Management, Boston University School of Public Health, Boston, Massachusetts; 2 Department of Health Services, Policy, and Practice, Brown University School of Public Health, Providence Rhode Island; 3 Now with Medicare Payment Advisory Commission, Washington DC; 4 Kidney Research Institute, Division of Nephrology, Department of Medicine, University of Washington, Seattle; 5 Providence VA Medical Center, Providence, Rhode Island

## Abstract

**Importance:**

In January 2021, under the 21st Century Cures Act, Medicare beneficiaries with end-stage renal disease (ESRD) were permitted to enroll in private Medicare Advantage (MA) plans for the first time. In the first year of the Cures Act, there was a 51% increase in MA enrollment among beneficiaries with ESRD.

**Objective:**

To examine changes in MA enrollment among Medicare beneficiaries with ESRD in the first 2 years of the Cures Act and, among beneficiaries newly enrolled in MA in 2021, to assess the proportion of beneficiaries who switched MA contracts and how the characteristics of contracts changed.

**Design, Setting, and Participants:**

This cross-sectional, population-based time-trend study was conducted from January 2020 to December 2022. Eligible participants included Medicare beneficiaries with ESRD. Data analysis was conducted from August 2023 to March 2024.

**Exposure:**

Enrollment in Medicare during the first 2 years of the 21st Century Cures Act.

**Main Outcomes and Measures:**

The primary outcomes were enrollment in MA, switching between traditional Medicare (TM) and MA, and switching between MA contracts from 2021 to 2022.

**Results:**

There were 718 252 unique Medicare beneficiaries with ESRD between 2020 and 2022 (1 659 652 beneficiary-years). In 2022, there were 583 203 beneficiaries with ESRD (mean [SD] age, 64.9 [14.1] years, 245 153 female (42.0%); 197 988 Black [34.0%]; 47 912 Hispanic [8.2%]). The proportion of beneficiaries with ESRD who were enrolled in MA increased from 25.1% (118 601 of 472 234 beneficiaries) in January 2020 to 43.1% (211 896 of 491 611 beneficiaries) in December 2022. Increases in MA enrollment were larger in the first year of the Cures Act (12.6 percentage points [pp]; 95% CI 12.3-12.8 pp) compared with the second year (5.7 pp; 95% CI, 5.5-5.9 pp). Changes between December 2020 and December 2022 ranged between 49.3% for Asian or Pacific Islander beneficiaries (difference = 13.0 pp; 95% CI, 12.2-13.8 pp) and 207.2% for American Indian or Alaska Native beneficiaries (difference = 17.0 pp; 95% CI, 15.3-18.7 pp). Changes were high among partial dual-eligible (difference = 35.5 pp; 95% CI, 34.9-36.1 pp; 134.7% increase) and fully dual-eligible beneficiaries (difference = 22.8 pp, 95% CI, 22.5-23.1 pp; 98.0% increase). Among 53 366 beneficiaries enrolled in MA in 2021, 37 439 (70.2%) remained in their contract, 11 730 (22.0%) switched contracts, and 4197 (7.9%) switched to TM in 2022. Compared with the characteristics of MA enrollees with ESRD in 2021, those in 2022 were more likely to be in contracts with lower premiums and with a rating of 4.5 stars or higher.

**Conclusions and Relevance:**

In this cross-sectional time-trend study of Medicare beneficiaries with ESRD, MA enrollment continued to increase in the second year of the Cures Act, particularly among racially or ethnically minoritized individuals and dual eligible populations. These findings suggest need to monitor the equity of care for beneficiaries with ESRD as they enroll in managed care plans.

## Key Points

**Question:**

How did enrollment in Medicare Advantage (MA) change among Medicare beneficiaries with end-stage renal disease (ESRD) in the first 2 years of the 21st Century Cures Act, and were changes different by beneficiary race, ethnicity, or dual eligibility status?

**Findings:**

In this cross-sectional study of 718 252 Medicare beneficiaries, the proportion of beneficiaries with ESRD enrolled in MA increased by 71.7% between January 2020 and December 2022, and few beneficiaries switched from MA to traditional Medicare.

**Meaning:**

The findings of this cross-sectional study suggest that policymakers may need to monitor access to care for Medicare beneficiaries with ESRD who newly enrolled in MA plans.

## Teaser

This cross-sectional study analyzes changes in Medicare Advantage enrollment in the first 2 years of the 21st Century Cures Act among beneficiaries with end-stage renal disease (ESRD).

## Introduction

Kidney failure impacts more than 800 000 people in the US, and disproportionately impacts low-income or racially or ethnically minoritized individuals.^1,2,3,4^ People with kidney failure require in-center thrice-weekly dialysis sessions, daily home dialysis, or kidney transplants for survival. Historically, people with kidney failure qualify for Medicare coverage through its end-stage renal disease (ESRD) benefit, with coverage beginning in the fourth month of in-center hemodialysis treatment or at 90 days. Through the 21st Century Cures Act (hereafter referred to as the Cures Act), Medicare beneficiaries with ESRD were able to enroll in private Medicare Advantage (MA) plans for the first time (effective January 1, 2021).^5,6^ Prior to this date, Medicare beneficiaries with ESRD were only able to enroll in MA plans under few exceptions, such as developing incident kidney failure while enrolled in an MA plan, and were otherwise enrolled in traditional Medicare (TM).

In the first year of the Cures Act, the total proportion of Medicare beneficiaries with ESRD who were enrolled in MA increased from 24.8% to 37.4% between December 2020 and December 2021, a relative increase of 50.8% that exceeded the Center for Medicare & Medicaid Services (CMS) initial projections of 30%.^7,8^ Increases were the largest among beneficiaries who were Black, Hispanic, or had dual Medicare-Medicaid eligibility.^7^ Whether and how trends in MA enrollment among Medicare beneficiaries with ESRD changed in the second year of the Cures Act is unknown. For example, MA enrollment among Medicare beneficiaries with ESRD could have continued to grow, consistent with national trends of all Medicare beneficiaries.^9,10^ Considering Medicare beneficiaries with ESRD disproportionately have low incomes, the availability of MA plans with low premiums or without premiums, as well as with supplemental benefits, may drive enrollment in or disenrollment from MA plans.^2,11,12,13^ Alternatively, considering the substantial needs of people with kidney failure, beneficiaries with ESRD may find that MA contracts restrict access to care (eg, insufficient health care networks or requirements for prior authorization).^14,15,16^ Dissatisfied beneficiaries with ESRD may consequently change MA contracts or altogether disenroll from MA to TM.^17,18,19^

The objective of our study was to evaluate national changes in MA enrollment among Medicare beneficiaries with ESRD in the first 2 years of the Cures Act—including new MA enrollment and switches in MA contracts—overall and also by race, ethnicity, and dual Medicare-Medicaid eligibility. Among beneficiaries with ESRD who enrolled in MA in 2021, we assessed changes in enrollment type (eg, TM or MA) in 2022 and whether characteristics of MA contracts changed between 2021 and 2022.

## Methods

### Study Design, Population, and Data

This cross-sectional study was approved by the Brown University institutional review board, and the CMS Privacy Board approved the study protocol and waived the need for informed consent owing to use of deidentified data. The study follows the Strengthening the Reporting of Observational Studies in Epidemiology (STROBE) reporting guideline for cross-sectional studies. We conducted a cross-sectional time-trend study of MA enrollment among Medicare beneficiaries with ESRD in 50 US states and the District of Columbia between 2020 and 2022. Building upon previous work,^7^ our study population included all Medicare beneficiaries with prevalent or incident ESRD between 2020 and 2022. All beneficiaries either required long-term dialysis or received a transplant.

We first compared how enrollment type changed between 2020 and 2022 among beneficiaries with prevalent ESRD in 2020. Specifically, we compared characteristics of beneficiaries who switched into MA at any time following the implementation of Cures Act with those who remained in TM in our study period. We then assessed changes in enrollment among beneficiaries who newly enrolled in MA in 2021 and examined if they remained in their 2021 MA contract, switched MA contracts, or switched from MA to TM. Lastly, among beneficiaries who switched MA contracts between 2021 and 2022, we evaluated changes in MA contract characteristics.

We used data from the Medicare Master Beneficiary Summary File (MBSF), which includes beneficiary-level monthly enrollment for each part of the Medicare program and sociodemographic information. There is an ESRD indicator in the Medicare MBSF for beneficiaries who are entitled to Medicare benefits due to having ESRD. To characterize beneficiaries’ clinical conditions, we linked the MBSF with Medicare Cost and Utilization Segment and counted the number of chronic conditions that were reported for prevalent patients using the Chronic Conditions Data Warehouse algorithm. The MBSF includes a county-level identifier, which we used to include county-level MA penetration. For beneficiaries enrolled in MA, the MBSF includes a contract identifier, allowing linkage to other CMS MA contract files. We used Ideon network data, which include MA plan-level clinician directories, to examine the breadth of dialysis facilities in MA contracts.^15,16^

### Measures

Our primary outcome was monthly enrollment in MA vs TM, and in our time-trend analysis, we examined changes between January 2020 and December 2022. We defined beneficiaries who remained in TM as those who were enrolled in only TM between 2020 and 2022 and beneficiaries who switched to MA as those who were enrolled in MA for at least 1 month in 2021 or 2022.

In our analysis assessing changes in enrollment type among beneficiaries who were newly enrolled in MA in 2021, we assigned beneficiaries into 1 of 3 categories: stayed in the same MA contract, switched MA contracts, and switched from MA to TM. We assigned beneficiaries to the first contract in which they were enrolled each year.

Characteristics in the MBSF included age, sex, race and ethnicity, dual eligible status, Medicare Part D cost sharing, and Medicare Part D low-income subsidy status. Depending on beneficiary income, those dually eligible for Medicare and Medicaid can qualify for the full package of Medicaid benefits (ie, full-benefit dual-eligibles) or can qualify for financial assistance for certain Medicare costs from the state Medicaid program (ie, partial-benefit dual-eligibles). To better understand differences in MA enrollment by dual eligibility status, we categorized beneficiaries into 3 groups: never dual, at least 1 month partial dual (but not full dual in any month), and at least 1 month full dual. The race and ethnicity variable is based on an algorithm developed by Research Triangle Institute (RTI) International that classifies beneficiaries as Hispanic or Asian or Pacific Islander according to data from the Social Security Administration or whether the beneficiary has a first or last name that RTI International determined was likely Hispanic or Asian.^20^ Additional race and ethnicity categories included American Indian or Alaska Native, Black, White, unknown, and other (defined as categories not captured by Medicare race and ethnicity variables).

Building upon previous work, MA characteristics were at the contract level.^16^ MA contract characteristics included plan type, contract age, having 1 or more special needs plans, geographic reach (eg, single-state vs multistate), parent organization, star rating, monthly premium (measured in quintiles), for-profit status, and dialysis facility network breadth.^16^

### Statistical Analysis

#### Main Analysis

We report absolute changes in MA enrollment between December 2020 and December 2022, overall, by race, ethnicity, and by dual eligibility status. We calculated adjusted changes in MA enrollment, where linear probability models adjusted for beneficiary age, sex, and state of residence and the unit of analysis was the beneficiary-year. For analyses comparing beneficiary characteristics by enrollment type, we used Pearson χ^2^ tests for categorical variables and *t* tests for continuous variables. For our analysis examining changes in contract composition among beneficiaries who switched MA contracts, we used bivariable linear probability regression models. Statistical significance was set at a 2-sided *P* < .05. Analyses were conducted using Stata version 17.0 (Stata Corp). Data analysis was conducted from August 2023 to March 2024.

#### Sensitivity Analyses

To assess the robustness of our results, we conducted multiple sensitivity analyses. First, we examined rates of MA enrollment for beneficiaries who became newly eligible for Medicare ESRD benefits or beneficiaries with incident ESRD in 2022 (only TM, only MA, or both TM and MA). Second, we present year-over-year changes in MA enrollment. Third, we assessed compositional shifts among Medicare beneficiaries with ESRD enrolled in MA for at least 1 month in 2021 vs 2022.

## Results

### Trends in MA Enrollment

A total of 718 252 unique Medicare beneficiaries with prevalent and incident ESRD in our study period were included (1 659 652 beneficiary-years). In 2022, there were 583 203 beneficiaries with ESRD (mean [SD] age, 64.9 [14.1] years, 245 153 female (42.0%); 197 988 Black [34.0%]; 47 912 Hispanic [8.2%]). The proportion of Medicare beneficiaries with ESRD who were enrolled in MA increased from 25.1% (118 601 of 472 234 beneficiaries) in January 2020 to 43.1% (211 896 of 491 611 beneficiaries) in December 2022, a 71.7% relative increase (Figure, A). In December 2020, 123 114 of 496 551 Medicare beneficiaries with ESRD (24.8%) were enrolled in MA. MA enrollment rates were comparable for Asian or Pacific Islander (6766 of 25 598 beneficiaries [26.4%]), Black (42 926 of 172 766 beneficiaries [24.8%]), Hispanic (25 121 of 86 974 beneficiaries [28.9%]), and White (45 090 of 190 760 beneficiaries [23.6%]) beneficiaries, but lower among American Indian or Alaska Native beneficiaries (502 of 6117 [8.2%]) (Figure, B). Between December 2020 and December 2022, increases in MA enrollment were substantial overall (18.3 percentage points [pp]) and for all racial and ethnic groups: American Indian or Alaska Native (difference = 17.0 pp; 95% CI, 15.3-18.7 pp; 207.2% relative increase), Asian or Pacific Islander (difference = 13.0 pp; 95% CI, 12.2-13.8 pp; 49.3% relative increase), Black (difference = 25.9 pp; 95% CI, 25.6-26.2 pp; 104.4% relative increase), Hispanic (difference = 18.2 pp; 95% CI, 17.8-18.6 pp; 62.9% relative increase), and White (difference = 12.7 pp; 95% CI, 12.4-13.0 pp; 53.6% relative increase). Increases between December 2020 and December 2022 were substantial for beneficiaries who were never dual (difference = 12.1 pp; 95% CI, 11.9-12.4 pp; 47.6% relative increase), partial dual-eligibles (difference = 35.5 pp; 95% CI, 34.9-36.1 pp; 134.7% relative increase), and full dual-eligibles (difference = 22.8 pp; 95% CI, 22.5-23.1 pp; 98.0% relative increase) (Figure, C). The magnitude of change was larger in the first year of the Cures Act (difference = 12.6 pp; 95% CI, 12.3-12.8 pp; 50.8% relative increase) compared with the second year (difference = 5.7 pp; 95% 5.5-5.9 pp; 15.3% relative increase) both overall and for all groups (eTable 1 in Supplement 1).

**Figure.**
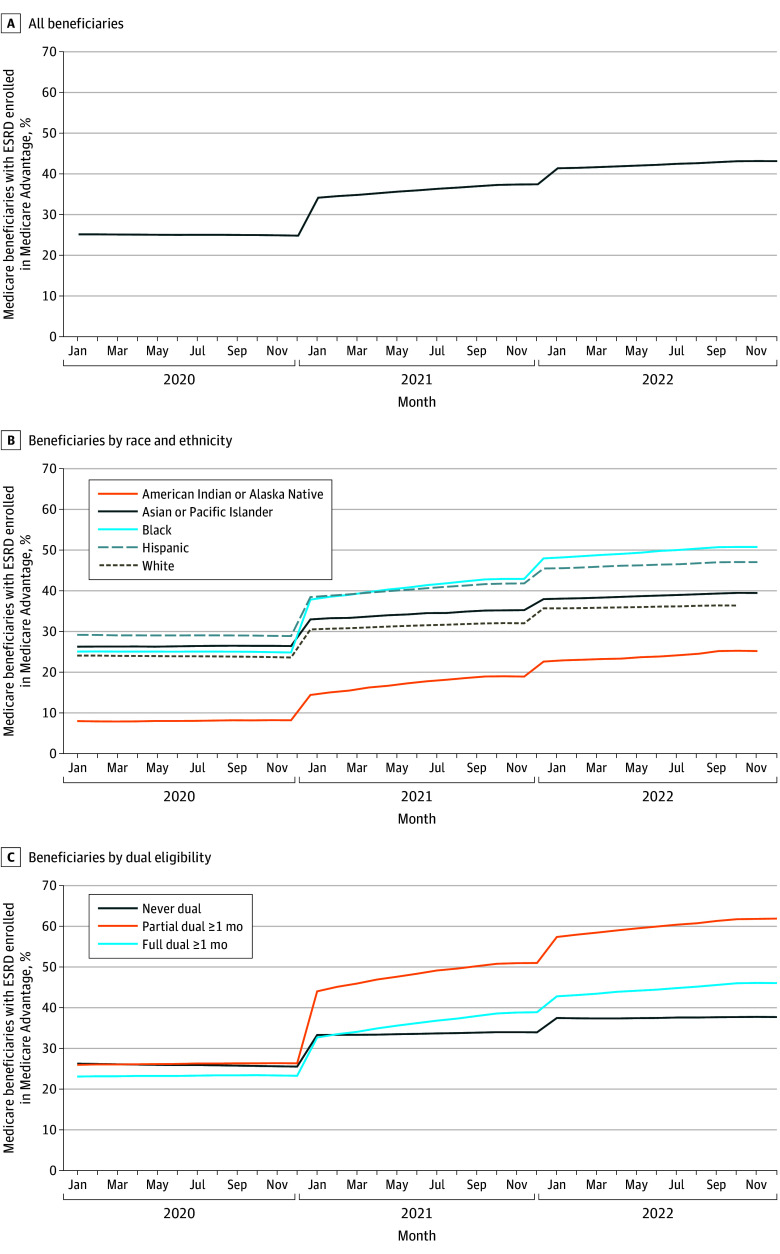
Proportion of Medicare Beneficiaries with End-Stage Renal Disease (ESRD) Enrolled in Medicare Advantage (MA), January 2020 to December 2022 In panel A, the numerator is the monthly number of all Medicare beneficiaries with ESRD who were enrolled in an MA plan. The denominator was the monthly number of all Medicare beneficiaries with ESRD. In panel B, the numerator is the race and ethnicity–specific monthly number of Medicare beneficiaries with ESRD who were enrolled in an MA plan. The denominator was the race and ethnicity–specific monthly number of Medicare beneficiaries with ESRD. In panel C, the numerator is the dual eligibility category–specific monthly number of Medicare beneficiaries with ESRD who were enrolled in an MA plan. The denominator is the dual eligibility category–specific number of Medicare beneficiaries with ESRD.

### Comparisons of Beneficiaries Who Switched From TM to MA

A total of 101 833 beneficiaries with prevalent ESRD in 2020 switched from TM to MA in 2021 or 2022 (Table 1). In our comparison of beneficiaries who remained in TM (270 080 beneficiaries) with those who switched to MA, 50 160 beneficiaries (49.3%) of those who switched to MA were Black compared with 81 192 beneficiaries (30.1%) who stayed in TM. Of the beneficiaries who switched to MA, 73 785 (72.5%) were full dual-eligibles, which was larger than those who stayed in TM (125 994 beneficiaries [46.7%]). Among beneficiaries who switched to MA, enrollment was highest in contracts that had 1 or more special needs plans (SNPs; 75 541 beneficiaries [79.8%]) or were part of the UnitedHealth Group parent organization (31 837 beneficiaries [33.6%]). Unadjusted and adjusted estimates of changes in MA enrollment overall and by race, ethnicity, and dual eligibility are presented in Table 2.

**Table 1.** Characteristics of Medicare Beneficiaries With Prevalent End-Stage Renal Disease Based on Enrollment Type, 2020 to 2022^a^

Characteristic	Beneficiaries, No. (%) (N = 371 913)^b^	
	Switched from traditional Medicare to Medicare Advantage (n = 101 833)	Remained in traditional Medicare (n = 270 080)
Beneficiary characteristics^c^		
Age, mean (SD), y	60.4 (12.6)	64.1 (14.9)
Sex		
Female	43 516 (42.7)	109 897 (40.7)
Male	58 317 (57.3)	160 183 (59.3)
Race and ethnicity^d^		
American Indian or Alaska Native	1180 (1.2)	4635 (1.7)
Asian or Pacific Islander	3604 (3.5)	12 752 (4.7)
Black	50 160 (49.3)	81 192 (30.1)
Hispanic	10 081 (9.9)	22 230 (8.2)
White	32 392 (31.8)	133 756 (49.5)
Unknown	1892 (1.9)	7386 (2.7)
Other^e^	2524 (2.5)	8129 (3.0)
Dual eligibility		
Never full or partial dual	2431 (2.4)	71 056 (26.3)
At least 1 mo partial dual	25 617 (25.2)	73 030 (27.0)
At least 1 mo full dual	73 785 (72.5)	125 994 (46.7)
Part D cost sharing		
No Part D coverage	32 999 (32.4)	154 420 (57.2)
Part D coverage 1-11 mo	17 285 (17.0)	19 656 (7.3)
Part D coverage 12 mo	51 512 (50.6)	95 917 (35.5)
Part D low-income subsidy		
No Part D cost sharing subsidy	23 878 (23.4)	72 882 (27.0)
Some Part D cost sharing subsidy	75 526 (74.2)	126 146 (46.7)
Unknown	2429 (2.4)	71 052 (26.3)
No. of chronic conditions, mean (SD)		
Ever^f^	6.0 (3.4)	6.2 (3.6)
2020^g^	3.5 (3.3)	4.1 (3.3)
County-level Medicare Advantage penetration, mean (SD)	47.1 (11.5)	43.6 (13.3)
Contract characteristics		
Plan type		
HMO or HMO POS	58 305 (61.6)	NA
Local PPO	28 385 (30.0)	NA
Regional PPO	3324 (3.5)	NA
Others	4639 (4.9)	NA
Contract age ≤5 y	23 079 (24.4)	NA
Special needs plan in contract	75 541 (79.8)	NA
Multi-state	52 515 (55.5)	NA
Parent organization		
UnitedHealth Group	31 837 (33.6)	NA
Humana	20 051 (21.2)	NA
CVS	6709 (7.1)	NA
Centene	2345 (2.5)	NA
Anthem	8864 (9.4)	NA
Other	24 847 (26.3)	NA
Star rating, mean (SD)	4.1 (0.5)	NA
Premium quartile		
1 (lowest)	11 707 (12.4)	NA
2	44 419 (46.9)	NA
3	25 019 (26.4)	NA
4	6706 (7.1)	NA
For profit	83 813 (88.5)	NA
Network breadth in 2021, mean (SD)	71.1 (25.4)	NA

Abbreviations: HMO, health maintenance organization; NA, not applicable; POS, point of service; PPO, preferred provider organization.

^a^ Sample includes Medicare beneficiaries with prevalent end-stage renal disease and enrolled in traditional Medicare in 2020, and therefore excludes beneficiaries with incident end-stage renal disease in 2021 or 2022 or beneficiaries who had Medicare Advantage in 2020.

^b^ *P* < .01 for all comparisons using Pearson χ^2^ tests for categorical variables and *t* tests for continuous variables.

^c^ Unless otherwise noted (ie, network breadth or number of chronic conditions), characteristics are from the most recent year of data for the beneficiary.

^d^ Race and ethnicity are from the Medicare Master Beneficiary Summary File, which uses an algorithm developed by the Research Triangle Institute (RTI) International to classify beneficiaries as Hispanic or Asian or Pacific Islander according to data from the Social Security Administration or whether they have a first or last name that RTI International determined was likely Hispanic or Asian in origin.

^e^ Other race includes categories not captured by Medicare race and ethnicity variables.

^f^ Chronic conditions (ever) included diagnosis of any of the following: acute myocardial infarction; arthritis; brain damage; breast cancer, prostate cancer, or other cancers; heart failure; chronic kidney disease; chronic obstructive pulmonary disease; cirrhosis; cystic fibrosis; depression or bipolar disorder; diabetes; drug or alcohol dependence; HIV or AIDS; hip fracture; ischemic heart disease; leukemia; multiple sclerosis; muscular dystrophy; obesity; pressure ulcers; spinal cord injury; and vascular disease.

^g^ Chronic conditions (2020) included the previous list as well as Parkinson disease and pneumonia.

**Table 2.** Changes in Medicare Advantage Enrollment Between December 2020 and December 2022^a^

Characteristic	Enrollment rate December 2020, No (%)	Change from December 2020 to December 2022, percentage point difference (95% CI)^b^	
		Unadjusted	Adjusted^c^
Overall	123 114 (24.8)	18.3 (18.1-18.5)	18.0 (17.8-18.2)
Race and ethnicity^d^			
American Indian or Alaska Native	502 (8.2)	17.0 (15.3-18.7)	16.9 (15.3-18.6)
Asian or Pacific Islander	6766 (26.4)	13.0 (12.2-13.8)	12.7 (12.0-13.5)
Black	42 926 (24.8)	25.9 (25.6-26.2)	25.5 (25.2-25.8)
Hispanic	25 121 (28.9)	18.2 (17.8-18.6)	17.9 (17.5-18.3)
White	45 090 (23.6)	12.7 (12.4-13.0)	12.3 (15.3-18.6)
Dual eligibility^e^			
Never dual	69 291 (25.5)	12.1 (11.9-12.4)	11.6 (11.3-11.8)
At least 1 mo partial dual	12 902 (26.4)	35.5 (34.9-36.1)	34.7 (34.1-35.2)
At least 1 mo full dual	40 917 (23.2)	22.8 (22.5-23.1)	22.7 (22.4-23.0)

^a^ The study sample includes Medicare beneficiaries with prevalent and incident end-stage renal disease. The outcome is beneficiary enrollment in Medicare Advantage in December. The unit of analysis is the beneficiary-year.

^b^ Differences and 95% CIs calculated using Margins command in Stata version 17.0 (StataCorp).

^c^ Covariates for adjusted changes were age (<18 years, 18-64 years, or ≥65 years), sex, and state of residence.

^d^ Models measuring changes by race or ethnicity include an indicator for race or ethnicity, year (2020 vs 2022), and their interaction (ie, race and ethnicity × year).

^e^ Models measuring changes by dual eligibility include an indicator for dual eligibility (never dual, at least 1 month partial dual, or at least 1 month full dual), year (2020 vs 2022), and their interaction (dual eligibility × year).

### Changes in Enrollment Type and Contract Characteristics Among Beneficiaries Who Newly Enrolled in MA in 2021 and Remained in MA in 2022

Among 53 366 beneficiaries who newly enrolled in MA in 2021, 37 439 (70.2%), remained in the same MA contract in 2022, 11 730 (22.0%) switched MA contracts, and 4197 (7.9%) switched to TM (Table 3). Of the 11 730 beneficiaries who switched MA contracts, 7101 (60.5%) were Black, which was larger than the proportion of beneficiaries who were Black and remained in their MA contract (18 648 of 37 439 beneficiaries [49.8%]) or switched to TM (2022 of 4197 beneficiaries [48.2%]).

**Table 3.** Changes in Medicare Enrollment Between 2021 and 2022 Among Beneficiaries With Prevalent End-Stage Renal Disease and Enrolled in MA in 2021^a^

Characteristic	Beneficiaries, No. (%) (N = 53 366)^b^		
	Remained in MA contract (n = 37 439)	Switched MA contracts (n = 11 730)	Switched from MA to traditional Medicare (n = 4197)
Beneficiary characteristic			
Age, mean (SD), y	59.6 (12.4)	60.3 (11.5)	61.3 (13.2)
Sex			
Female	15 743 (42.0)	5036 (42.9)	1865 (44.4)
Male	21 696 (58.0)	6694 (57.1)	2332 (55.6)
Race and ethnicity			
American Indian or Alaska Native	403 (1.1)	122 (1.0)	68 (1.6)
Asian or Pacific Islander	1326 (3.5)	302 (2.6)	152 (3.6)
Black	18 648 (49.8)	7101 (60.5)	2022 (48.2)
Hispanic	3853 (10.3)	927 (7.9)	475 (11.3)
White	11 561 (30.9)	2889 (24.6)	1263 (30.1)
Other^c^	708 (1.9)	162 (1.4)	81 (1.9)
Unknown	940 (2.5)	227 (1.9)	136 (3.2)
Dual eligibility			
Never full or partial dual	12 454 (33.3)	3205 (27.3)	1382 (32.9)
At least 1 mo partial dual	7148 (19.1)	2607 (22.2)	600 (14.3)
At least 1 mo full dual	17 834 (47.6)	5916 (50.4)	2214 (52.8)
Part D low-income subsidy			
No Part D cost sharing subsidy	8963 (23.9)	2138 (18.2)	718 (17.1)
Some Part D cost sharing subsidy	27 834 (74.3)	9445 (80.5)	3079 (73.4)
Unknown	642 (1.7)	147 (1.3)	400 (9.5)
Contract characteristics, 2022			
Plan type			
HMO or HMO POS	23 827 (63.6)	7283 (62.1)	NA
Local PPO	10 752 (28.7)	3854 (32.9)	NA
Regional PPO	1476 (3.9)	377 (3.2)	NA
Others	1384 (3.7)	215 (1.8)	NA
Contract age ≤5 y	8609 (23.0)	3692 (31.5)	NA
Special needs plan in contract	30 458 (81.4)	9992 (85.2)	NA
Multi-state	21 317 (57.0)	6704 (57.2)	NA
Parent organization			
UnitedHealth group	12 743 (34.0)	4474 (38.)	NA
Humana	8826 (23.6)	2327 (19.8%)	NA
CVS	2410 (6.4)	858 (7.3)	NA
Centene	1021 (2.7)	55 (0.5)	NA
Anthem	3245 (8.7)	1043 (8.9)	NA
Other	9194 (24.6)	2972 (25.3)	NA
Star rating			
≤3.5 Stars	6214 (17.0)	2103 (17.9)	NA
4-4.5 Stars	18 132 (49.7)	5647 (50.7)	NA
5 Stars	5254 (14.4)	1181 (10.6)	NA
Missing, plan too new to be measured, or not enough data available	6883 (18.9)	2205 (19.8)	NA
Premium quartile			
1 (lowest)	4720 (12.6)	1716 (14.6)	NA
2	19 046 (50.9)	5876 (50.)	NA
3	9014 (24.1)	3477 (29.6%)	NA
4	2903 (7.8)	439 (3.7)	NA
For profit	33 064 (88.3)	10 966 (93.5)	NA
County-level MA penetration, mean (SD)	47.8 (11.3)	47.5 (10.9)	45.8 (12.1)

Abbreviations: HMO, health maintenance organization; MA, Medicare Advantage; NA, not applicable; POS, point of service; PPO, preferred provider organization.

^a^ Sample is limited to beneficiaries who were newly enrolled in MA in 2021 and survived at least 1 month into 2022.

^b^ *P* < .001 for all comparisons using Pearson χ^2^ tests for categorical variables and *t* tests for continuous variables for all comparisons.

^c^ Other race includes categories not captured by Medicare race and ethnicity variables.

Among 49 169 beneficiaries enrolled in MA in 2021 and 2022, 11 730 (23.9%) switched contracts between the 2 years. There were statistically significant differences in the characteristics of MA contracts in 2021 vs 2022, including increases in the proportion of beneficiaries enrolled in MA plans that were of a contract age of 5 years or less (difference = 3.3 pp; 95% CI, 2.2-4.5 pp), part of the UnitedHealth Group parent organization (difference = 9.8 pp; 95% CI, 8.6-11.0 pp), had star ratings of 4.5 or higher (difference = 14.9 pp; 95% CI, 13.9-15.9 pp), and were in the lowest quartiles of premiums (quartile 1, difference = 1.4 pp; 95% CI, 0.5-2.3 pp; quartile 2, difference = 7.2 pp, 95% CI, 5.9-8.5 pp) (Table 4).

**Table 4.** MA Contract Characteristics Among Beneficiaries With End-Stage Renal Disease Who Stayed Enrolled in MA but Switched Contracts Between 2021 and 2022 (N = 11 730)^a^

Characteristic	Percentage of beneficiaries, mean (SD)		Unadjusted difference, percentage points (95% CI)	*P* value^b^
	2021	2022		
Plan type				
HMO or HMO POS	63.4 (48.2)	62.1 (48.5)	−1.3 (−2.6 to −0.1)	.04
Local PPO	27.5 (44.6)	32.9 (47.0)	5.4 (4.2 to 6.5)	<.001
Regional PPO	6.3 (24.4)	3.2 (17.6)	−3.1 (−3.7 to −2.6)	<.001
Other	2.8 (16.4)	1.8 (13.4)	−0.9 (−1.3 to −0.5)	<.001
Contract age, y				
≤5	28.1 (45.0)	31.5 (46.4)	3.3 (2.2 to 4.5)	<.001
6-10	14.6 (35.3)	13.2 (33.9)	−1.3 (−2.3 to −0.5)	.003
≥10	56.5 (49.6)	54.2 (49.8)	−2.3 (−3.6 to −1.0)	<.001
Any special needs plan	80.6 (39.6)	85.2 (35.5)	4.6 (3.7 to 5.6)	<.001
Multi-state	59.7 (49.1)	57.2 (49.5)	−2.5 (−3.8 to−1.3)	<.001
Parent organization				
UnitedHealth Group	28.4 (45.1)	38.1 (48.6)	9.8 (8.6 to 11.0)	<.001
Humana	27.5 (44.7)	19.8 (39.9)	−7.7 (−8.8 to −6.6)	<.001
CVS	6.9 (25.4)	7.3 (26.0)	0.4 (−0.2 to 1.1)	.22
Centene	0.5 (7.1)	0.5 (6.8)	−0.0 (−0.2 to 0.1)	.64
Anthem	9.6 (29.4)	8.9 (28.5)	−0.7 (−1.4 to 0.1)	.08
Other	27.1 (44.5)	25.3 (43.5)	−1.8 (−2.9 to −0.7)	.002
Star rating ≥4.5	10.9 (31.2)	25.8 (43.8)	14.9 (13.9 to 15.9)	<.001
Premium quartile				
1 (lowest)	13.2 (33.9)	14.6 (35.3)	1.4 (0.5 to 2.3)	.002
2	42.9 (49.5)	50.1 (50.0)	7.2 (5.9 to 8.5)	<.001
3	28.9 (45.3)	29.6 (45.7)	0.8 (−0.4 to 2.0)	.18
4	2.8 (16.5)	3.7 (19.0)	0.9 (0.49 to 1.4)	<.001
For profit	95.9 (19.9)	95.2 (21.5)	0.7 (0.2 to 1.3)	.008
Narrow network breadth in 2021	6.7 (24.9)	7.0 (25.6)	0.4 (−0.3 to 1.0)	.26

Abbreviations: HMO, health maintenance organization; MA, Medicare Advantage; POS, point of service; PPO, preferred provider organization.

^a^ Sample is limited to individuals who stayed in MA in 2021 and 2022 but were in different contracts in each year.

^b^ Statistical significance determined using bivariable linear probability models.

### Sensitivity Analyses

Among 127 105 beneficiaries with no prior diagnosis of ESRD before 2022, 72 003 (56.7%) were only enrolled in TM, 44 563 (35.1%) were only enrolled in MA, and 10 539 (8.3%) were enrolled in both TM and MA (eTable 2 in Supplement 1). Relative year-over-year changes in MA enrollment indicated that changes in MA enrollment were larger in the first year relative to the second year (eTable 1 in Supplement 1). There were modest changes in the composition of beneficiaries enrolled in MA between 2021 and 2022 (eTable 3 in Supplement 1).

## Discussion

In this cross-sectional study using enrollment data for all Medicare beneficiaries with ESRD in the US, we found that MA enrollment continued to increase in the second year of the Cures Act. In the first 2 years of the Cures Act, there was a relative increase of 73.8% in MA enrollment nationwide among Medicare beneficiaries with ESRD. The largest relative increases in MA enrollment between December 2020 and December 2022 were among American Indian and Alaska Native, Black, Hispanic, and both partial and full dual-eligible beneficiaries. Most beneficiaries remained in the same Medicare insurance type (eg, TM or MA) in 2021 and 2022. Among beneficiaries with enrollment type switches between 2021 and 2022, rates of switching from TM to MA were higher than those from MA to TM, and the population switching MA plans were disproportionately comprised of Black patients. Finally, 23.9% of beneficiaries who stayed in MA switched their contract between 2021 and 2022. Compared with MA enrollees with ESRD in 2021, those in 2022 were more likely to be in a newer plan (<5 years), a plan with higher star ratings, a plan with lower premiums, and those owned by UnitedHealth Group.

To our knowledge, our study provides 3 new contributions to the literature. First, our work extends a previous study^7^ by evaluating changes in the first 2 years of the Cures Act. We found that MA enrollment among beneficiaries with ESRD continued to increase, although the magnitude of increase was more modest in the second year when compared with the first year. Second, we found substantially larger increases in MA enrollment among partial-benefit dual-eligibles. Third, we evaluated inertia in MA plan choices or continuous enrollment in the same MA contract in the first 2 years of the Cures Act and found that approximately one-fifth of beneficiaries who stayed in MA changed contracts between years.

Our study has crucial implications for the equity of care delivered to beneficiaries who are from racially or ethnically minoritized groups or have low incomes. Increases in MA enrollment among Medicare beneficiaries with ESRD continue to surpass the initial CMS projections (30% in 2021), and Black, Hispanic, and dually eligible (both full- and partial-benefit) beneficiaries enrolled in MA at disproportionately higher rates.^6,8^ Furthermore, Black beneficiaries who enrolled in MA in 2021 had substantially higher rates of switching to another plan in 2022, suggesting that some MA plans may not be meeting the needs of Black beneficiaries with ESRD. Although our findings align with national trends in MA enrollment (ie, not limited to beneficiaries with ESRD),^9,10,11,13^ beneficiaries with kidney failure often are more clinically complex than other Medicare beneficiaries and require consistent access to dialysis treatment. Prior to the Cures Act, MA contracts networks included, on average, 51% of dialysis facilities in their service areas, and beneficiaries with ESRD who were Hispanic, Asian or Pacific Islander, or American Indian or Alaska Native were more likely to be enrolled in MA contracts with narrow dialysis facilities networks compared with non-Hispanic White beneficiaries, potentially limiting access to care.^16^ Dialysis facility network breadth appeared to be wider in 2021, the first year of the Cures Act. As MA enrollment continues to increase—particularly for beneficiaries from racially or ethnically minoritized groups—it is crucial to monitor adequacy of dialysis facility networks in MA contracts. Additional research on reasons for increased MA enrollment among dually eligible (both full- and partial-benefit) beneficiaries is needed. More broadly, assessing whether MA networks are equipped to facilitate access to care for increasingly more beneficiaries with ESRD will be important.

Second, voluntary disenrollment from an MA plan or changing MA plans may reflect dissatisfaction with an MA plan’s benefits (eg, network adequacy or out of pocket costs).^14,17^ Those who stayed enrolled in MA but switched contracts may have been dissatisfied with the plan they were enrolled in in 2021, but not necessarily with MA compared with TM. MA enrollees appeared to move out of plans with either low star ratings or plans too new to have a rating into contracts with higher star ratings, suggesting that beneficiaries accounted for quality ratings when selecting a new MA plan. Considering the increased number of new MA plans and the amount of advertising during MA open enrollment, beneficiaries may select plans with lower premiums or better benefits. Approximately three-quarters of our sample was enrolled among 4 insurers, and market share grew from 2021 to 2022—particularly for UnitedHealth Group—illustrating the high degree of consolidation in the current MA market. MA plans receive a capitated payment from CMS, and there are incentives to improve value of care or offer unique benefits that may appeal to beneficiaries (eg, medically tailored meals).^21^ Alternatively, contracts can develop networks that potentially limit access to care or discourage use of high-cost services.^15^ Furthermore, beneficiaries with ESRD may want to switch to TM but may be denied Medigap on the basis of preexisting conditions.^22^ As such, inertia may reflect inability to change from an MA plan. While many beneficiaries stayed in TM or MA in our study period, changes in contracts were at slightly higher rates than national averages.^14^ Additional research on reasons for disenrollment may identify whether some plan types, cost sharing arrangements, or benefit designs perform better for beneficiaries with ESRD.

### Limitations

Our study has several limitations. First, our race and ethnicity variable may misclassify individuals—particularly Hispanic or Asian people.^23^ Second, we were unable to assess from our data which beneficiaries received Medicare ESRD benefits because of long-term dialysis or receipt of a kidney transplant. Additional information on MA enrollment among the transplant population is needed. Third, it is possible some people were excluded from our sample because they had a diagnosis of kidney failure but had not yet received Medicare ESRD benefits. Fourth, there may be variation between benefits within contracts. Fifth, because we did not have data available on MA spending or CMS payments, we were unable to measure potential differences in out-of-pocket costs, which might play a role in MA enrollment. Sixth, we were unable to assess potential mechanisms or reasons that beneficiaries with ESRD may have switched MA contracts or disenrolled from MA to TM. We also did not include data on other outcomes like quality of care. Seventh, our study period included the first years of the COVID-19 pandemic, which may have impacted our outcomes. Specifically, previous work indicates that the COVID-19 pandemic impacted treatment initiation for incident kidney failure and mortality rates among people with kidney failure, with disproportionate impacts on Black patients.^7,24^ While mortality rates could differ for TM vs MA beneficiaries with ESRD, historically there has not been evidence that mortality rates are significantly different between the 2 groups.^25^

## Conclusions

In this cross-sectional time-trend study, we found that among Medicare beneficiaries with ESRD benefits, MA enrollment continued to increase in the first 2 years of the Cures Act, albeit relatively smaller in magnitude compared with the first year. Black, Hispanic, and American Indian or Alaska Native beneficiaries and those with any dual Medicare-Medicaid coverage continued to enroll MA plans at substantially higher rates in the second year of the Cures Act, highlighting the need to monitor the equity of care for patients with kidney failure as they transition to managed care.

## Notes

Supplement 1. **eTable 1.** Relative Year-Over-Year Changes in MA Enrollment **eTable 2.** Medicare Advantage Enrollment Among Beneficiaries With Incident ESRD Benefits in 2022 **eTable 3.** Characteristics of Medicare Beneficiaries With End-Stage Renal Disease With at Least One Month Enrolled in Medicare Advantage by Year

Supplement 2.
Data Sharing Statement
