# Supplementary material for: Medicare Advantage Enrollment Following the 21st Century Cures Act in Adults With End-Stage Renal Disease
Source: JAMA Netw Open. 2024 Sep 12;7(9):e2432772. doi: 10.1001/jamanetworkopen.2024.32772 (PMC11393715; doi:10.1001/jamanetworkopen.2024.32772)
Supplement: Supplement 1. — eTable 1. Relative Year-Over-Year Changes in MA Enrollment eTable 2. Medicare Advantage Enrollment Among Beneficiaries With Incident ESRD Benefits in 2022 eTable 3. Characteristics of Medicare Beneficiaries With End-Stage Renal Disease With at Least One Month Enrolled in Medicare Advantage by Year [file jamanetwopen-e2432772-s001.pdf]

Supplemental Online Content

Nguyen KH, Oh EG, Meyers DJ, et al. Medicare Advantage enrollment following the 21st century cures act in adults with end-stage renal disease. *JAMA Netw. Open*. 2024;7(9):e2432772. doi:10.1001/jamanetworkopen.2024.32772

eTable 1. Relative Year-Over-Year Changes in MA Enrollment

eTable 2. Medicare Advantage Enrollment Among Beneficiaries With Incident ESRD Benefits in 2022

eTable 3. Characteristics of Medicare Beneficiaries With End-Stage Renal Disease With at Least One Month Enrolled in Medicare Advantage by Year

This supplemental material has been provided by the authors to give readers additional information about their work.

© 2024 Nguyen KH et al. *JAMA Network Open*.

eTable 1. Relative Year-Over-Year Changes in MA Enrollment

	MA Enrollment Rate, Dec 2020 (%)	Dec 2020-Dec 2022 (Relative %)	Dec 2020-Dec 2021 (Relative %)	Dec 2021-Dec 2022 (Relative %)
Overall	24.8	73.8	50.8	15.3%
**Race or Ethnicity**				
American Indian or Alaska Native	8.2	207.2	130.8	33.1
Asian/Pacific Islander	26.4	49.3	33.4	11.9
Black	24.8	104.4	72.8	18.3
Hispanic or Latino	28.9	62.9	44.8	12.4
White	23.6	53.6	35.5	13.3
**Dual Eligibility**				
Never Dual	25.5	47.6	32.9	11.1
At Least One Month Partial Dual	26.4	134.7	93.4	21.3
At Least One Month Full Dual	23.2	98.0	67.3	18.3

Notes. MA refers to “Medicare Advantage”

© 2024 Nguyen KH et al. *JAMA Network Open*.

eTable 2. Medicare Advantage Enrollment Among Beneficiaries With Incident ESRD Benefits in 2022

	**Incident TM (n=72,003)**	**Incident MA (n=44,563)**	**Incident Mixed (n=10,539)**
Overall	56.7%	35.1%	8.3%

Notes. ESRD refers to “End-Stage Renal Disease.” TM refers to “Traditional Medicare.” MA refers to “Medicare Advantage”

© 2024 Nguyen KH et al. *JAMA Network Open*.

eTable 3. Characteristics of Medicare Beneficiaries With End-Stage Renal Disease With at Least One Month Enrolled in Medicare Advantage by Year

	2021 (n=223,182)	2022 (n=260,386)	Total (n=483,568)	*p*
**Months of Medicare Advantage (MA) Coverage**	10.0 (3.4)	10.20 (3.3)	10.1 (3.3)	<0.001
**Part D Enrollment, %**	176,457 (98.5%)	201,998 (98.4%)	378,455 (98.4%)	0.016
**Age (years), Mean(SD)**	66.9 (12.8)	66.5 (12.8)	66.7 (12.8)	<0.001
**Female**	98,829 (44.3%)	115,016 (44.2%)	213,845 (44.2%)	0.441
**Race or Ethnicity**				
American Indian or Alaska Native	1,399 (0.6%)	1,879 (0.7%)	3,278 (0.7%)	<0.001
Asian/Pacific Islander	8,809 (3.9%)	10,633 (4.1%)	19,442 (4.0%)	
Black	87,862 (39.4%)	104,464 (40.1%)	192,326 (39.8%)	
Hispanic	17,750 (8.0%)	22,057 (8.5%)	39,807 (8.2%)	
White	96,306 (43.2%)	109,213 (41.9%)	205,519 (42.5%)	
Other	7,211 (3.2%)	7,022 (2.7%)	14,233 (2.9%)	
Unknown	3,845 (1.7%)	5,118 (2.0%)	8,963 (1.9%)	
**Original Reason for Medicare Eligibility**				
Old Age and Survivor's Insurance (OASI)	88,811 (39.8%)	100,204 (38.5%)	189,015 (39.1%)	<0.001
Disability Insurance Benefits (DIB)	70,257 (31.5%)	82,591 (31.7%)	152,848 (31.6%)	
End-Stage Renal Disease (ESRD)	43,692 (19.6%)	56,356 (21.6%)	100,048 (20.7%)	
DIB and ESRD	20,422 (9.2%)	21,235 (8.2%)	41,657 (8.6%)	
**Current Reason for Medicare Eligibility**				
OASI	139,479 (62.5%)	156,363 (60.1%)	295,842 (61.2%)	<0.001
DIB	57,722 (25.9%)	62,710 (24.1%)	120,432 (24.9%)	
ESRD	18,353 (8.2%)	25,784 (9.9%)	44,137 (9.1%)	
DIB and ESRD	7,628 (3.4%)	15,529 (6.0%)	23,157 (4.8%)	
**Dual Eligibility**				
Never Dual	109,570 (49.1%)	120,683 (46.4%)	230,253 (47.6%)	<0.001
At Least One Month Partial Dual	28,888 (12.9%)	34,034 (13.1%)	62,922 (13.0%)	
At Least One Month Full Dual	84,650 (37.9%)	105,652 (40.6%)	190,302 (39.4%)	
**Part D Cost Sharing**				
No Part D Coverage	3,489 (1.6%)	4,312 (1.7%)	7,801 (1.6%)	<0.001
Part D Coverage 1-11 Months	46,373 (20.8%)	51,705 (19.9%)	98,078 (20.3%)	
Part D Coverage 12 Months	173,320 (77.7%)	204,369 (78.5%)	377,689 (78.1%)	
**Part D Low Income Subsidy**				

© 2024 Nguyen KH et al. *JAMA Network Open*.

No Part D Cost Sharing Subsidy	92,148 (41.3%)	101,865 (39.1%)	194,013 (40.1%)	<0.001
Some Part D Cost Sharing Subsidy	127,547 (57.1%)	154,211 (59.2%)	281,758 (58.3%)	
Unknown	3,487 (1.6%)	4,310 (1.7%)	7,797 (1.6%)	
**County-Level MA Penetration, Mean(SD)**	46.236 (11.435)	48.893 (11.112)	47.667 (11.340)	<0.001

© 2024 Nguyen KH et al. *JAMA Network Open*.
