# Supplementary material for: Medicare Advantage Enrollment Following the 21st Century Cures Act in Adults With End-Stage Renal Disease
Source: JAMA Netw Open. 2024 Sep 12;7(9):e2432772. doi: 10.1001/jamanetworkopen.2024.32772 (PMC11393715; doi:10.1001/jamanetworkopen.2024.32772)
Supplement: Supplement 2. — Data Sharing Statement [file jamanetwopen-e2432772-s002.pdf]

Data Sharing Statement

Nguyen. Medicare Advantage Enrollment Following the 21st Century Cures Act in Adults With End-Stage Renal Disease. *JAMA Netw Open*. Published September 12, 2024. doi:10.1001/jamanetworkopen.2024.32772

Data
Data available: No
